# Insights into the Evolution of a Cryptic Radiation of Bats: Dispersal and Ecological Radiation of Malagasy *Miniopterus* (Chiroptera: Miniopteridae)

**DOI:** 10.1371/journal.pone.0092440

**Published:** 2014-03-18

**Authors:** Les Christidis, Steven M. Goodman, Kate Naughton, Belinda Appleton

**Affiliations:** 1 National Marine Science Centre, Southern Cross University, Coffs Harbour, New South Wales, Australia; 2 Department of Genetics, University of Melbourne, Melbourne, Victoria, Australia; 3 Field Museum of Natural History, Chicago, Illinois, United States of America; 4 Association Vahatra, BP 3972, Antananarivo, Madagascar; 5 Life and Environmental Science, Deakin University, Victoria, Australia; Università degli Studi di Napoli Federico II, Italy

## Abstract

The past decade has seen a proliferation of new species of *Miniopterus* bats (family Miniopteridae) recognized from Madagascar and the neighboring Comoros archipelago. The interspecific relationships of these taxa, their colonization history, and the evolution of this presumed adaptive radiation have not been sufficiently explored. Using the mitochondrial cytochrome-*b* gene, we present a phylogeny of the Malagasy members of this widespread Old World genus, based on 218 sequences, of which 82 are new and 136 derived from previous studies. Phylogenetic analyses recovered 18 clades, which divide into five primary lineages: (1) *M*. *griveaudi*; (2) *M*. *mahafaliensis*, *M*. *sororculus* and X3; (3) *M*. *majori*, *M*. *gleni* and *M*. *griffithsi*; (4) *M*. *brachytragos*; *M*. *aelleni*A, and *M*. *aelleni*B; and (5) *M*. *manavi* and *M*. *petersoni* recovered as sister species, which were in turn linked to a group comprising *M*. *egeri* and five genetically distinct populations referred to herein as P3, P4, P5, P6 and P7. Beast analysis indicated that the initial divergence within the Malagasy *Miniopterus* radiation took place 4.5 Myr; most species diverged between 4 and 2.5 Myr, and a secondary period was between 1.25 and 1 Myr. DNA K2P-distances between recognized taxa ranged from 12.9% to 2.5% and intraspecific variation was less than 1.8%. Of the 18 identified clades, Latin binomials are only associated with 11, which indicates much greater differentiation than currently recognized for Malagasy *Miniopterus*. These data are placed in a context of the dispersal history of this genus on the island and patterns of ecological diversity.

## Introduction

Madagascar is well known as a center of endemism for a wide assortment of plant and animal taxa. This is directly associated with the island's considerable ecological and topographic diversity, as well as isolation in deep geological time [1], [2], [3]. In contrast to other areas of the Old World tropics, Madagascar's distinctive biota contains numerous endemic groups at higher taxonomic levels, representing distinct radiations. In some cases, such as certain reptiles [4], these endemic groups are best explained as vicariant relicts originating before the break-up of Gondwana some 165 million years ago [5], [6]. However, much more common are plant and animal groups that successfully colonized the island by over-water dispersal in more recent geological time [7]. These post-Gondwana-split colonizations occurred across multiple geological periods, resulting in levels of differentiation ranging from endemic orders to genera [7]–[9].

Recent molecular research has provided considerable new insight into these different evolutionary events, levels of taxonomic diversity, and the complexity of various Malagasy radiations. These studies have uncovered cryptic species belonging to previously unrecognized taxa that are largely indiscernible using more classic taxonomic characters. As such, the results of these studies provide the means to differentiate shared evolutionary history versus convergence. The recent recognition of an endemic Malagasy bird family, the Bernieridae, is an excellent example. It comprises 11 species that share no defining morphological characters and formerly were placed in three different songbird families [10], [11]. Members of the endemic family Vangidae were also previously placed in three separate songbird families [12], [13]. Finally, although the island holds a considerable diversity of land mammals, all existing groups (carnivorans, lemurs, rodents, and tenrecs), which show extraordinary morphological variation, can be explained by four colonization events [14]. Study of the extant fauna has therefore shown that successful colonization of Madagascar by land mammals has been rare and accompanied by subsequent adaptive radiations. While several different hypotheses have been presented to explain patterns of endemism and micro-endemism in the island's biota [2], [3], [15], recent research has shown that a single model cannot explain the different patterns observed in the living biota of the island.

In the present study, we explore the complex micro-radiation of a widespread Old World group of bats, the family Miniopteridae. While their wing structure is not designed for high speed, they are relatively strong flyers [16], attested by their capacity to colonize offshore and oceanic islands. In a review of Madagascar's chiropteran fauna, Peterson et al. [17] reported four species from the island: one endemic, two shared with the nearby Comoro archipelago, and one in common with continental Africa. Less than two decades later, largely based on insights from molecular genetics and to a lesser extent morphology and bioacoustics, 11 species are recognized today from the island, all endemic with the exception of two shared with the Comoros [18], [19].

To date, systematic research on Malagasy *Miniopterus* has concentrated on the delimitation of species. Little attention has been given to the evolutionary relationships of the different taxa relative to Madagascar or nearby islands and continental areas. The purposes of this paper are to apply molecular phylogenetic data to explore primarily the patterns of diversification of members of this genus in Madagascar, within an ecological context. Secondarily, to explore aspects of their colonization history and patterns of dispersal.

## Methods

### Bat sampling and specimens examined

**Figure 1:**
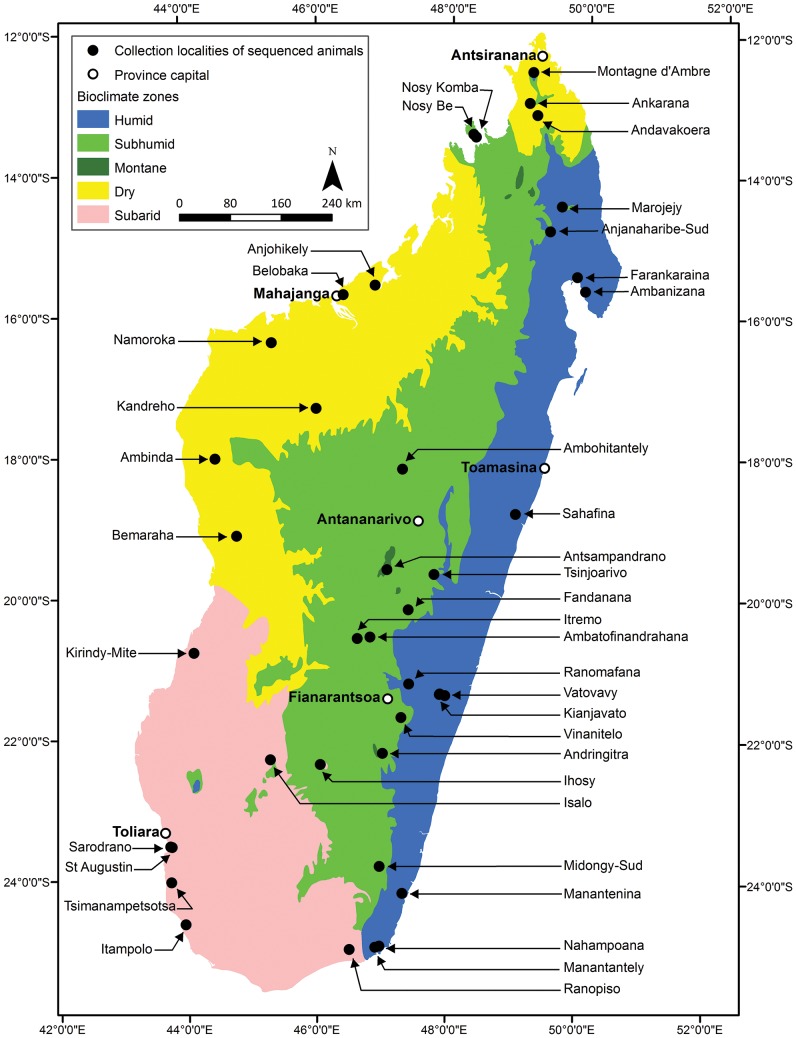
Bioclimatic map of Madagascar with collection localities of all specimens sequenced in this study (see Table S1).

Specimens were captured from diverse areas and habitats for this study, essentially covering the entire range of *Miniopterus* spp. on Madagascar (Figure 1), using mist nets and harp traps most often placed at cave entrances. This study was conducted in strict accordance with the terms of research permits issued by national authorities in Madagascar (Direction du Système des Aires Protégées, Direction Générale de l’Environnement et des Forêts, and Madagascar National Parks; and in the Union of the Comoros (Centre National de Documentation et de Recherche Scientifique), following the laws of these countries, and the associated research permit numbers are listed in the acknowledgements. Seventy-five animals were captured, manipulated and euthnanized in accordance with guidelines accepted by these different national authorities and the scientific community for the handling of wild animals [20]. Voucher specimens are housed in the Field Museum of Natural History (FMNH), Chicago, and the Université d’Antananarivo, Département de Biologie Animale (UADBA), Antananarivo.

The mitochondrial cytochrome-*b* (cyt-*b*: 1140 bp) has previously been shown to be informative at the species level in the study of miniopterine bats [21]–[24], which is our primary focus herein. The dataset we have employed includes all recently published work on Malagasy miniopterine species and incorporates new sequences from specimens previously defined as *M*. *manavi* [17]. In total, 264 sequences have been employed herein, 75 acquired for this study and 189 previously used in different taxonomic studies (Table S1). The dataset also incorporates sequences from islands in the Comoros, including Grande Comore and Anjouan [25]. Due to the reliance on pre-existing published sequences to build a complete taxonomy, the study was limited to the use of cyt-*b* alone, specifically as a number of tissue samples amomgst the 264 samples are not available to the authors for sequencing nuclear or microsatellite markers.

Cyt-*b* sequences of African, European, Asian and Australasian *Miniopterus* spp. were also included from Genbank records (Table S1). With no clear sister group to the genus *Miniopterus* or the family Miniopteridae, we chose *Myotis ricketti* (EF530349) as the outgroup. The use of outgroup sequences from other chiropteran families did not alter the relationships between the *Miniopterus* spp. [23], [24], [26], [27] Analysis using *M*. *ricketti* as the outgroup resulted in two fully supported (posterior probability 1.00) *Miniopterus* clades: one consisting of Malagasy, African and European taxa and another consisting of Asian and Australasian taxa. For reasons detailed below and to improve resolution, the Asian and Australasian clade was then used as the outgroup for determining relationships between the Malagasy, African and European taxa.

### Molecular analysis

Production of the sequences was achieved using the same methods described in previous studies on Malagasy *Miniopterus* [27].

Sequences were assembled and aligned using Sequencher version 4.6 (Gene Codes Corporation, Ann Arbor, MI). Analysis using DNA strider [28] showed that sequences did not contain insertions, deletions or stop codons. All new sequences were deposited in GenBank (Accession numbers listed in Table S1). The program jModeltest v2.1.4 [29], [30] reported HKY + G as the optimal nucleotide substitution model for the dataset according to Hierarchical Likelihood Ratio tests, Aikake Information Criterion and Bayesian Information Criterion. This model was applied to the Bayesian and molecular clock analyses.

Bayesian analyses were conducted using MrBayes v3.2 [31] under uniform priors. Four chains were run under MrBayes for 2,000,000 generations with a sampling frequency of 1,000. Burn-in was set at 25% of initial trees. The deviation of split frequencies was below 0.01 at the conclusion of the analysis. Maximum likelihood analyses were run using RaXML Black Box workbench [32], [33], using the GTRGAMMA model. Bootstrap values were estimated using 1000 pseudoreplicates.

Bayesian and ML analyses were initially run with the full dataset in order to confirm fine-scale topology (Figure S1); however, due to the influence of wide variations in sequence divergences on the gamma distribution and increased branch length, these analyses were repeated using only two to four sequences from each major clade and with the removal of the highly divergent outgroup. *Myotis* proved to be more than 24% divergent in cyt-*b* (Kimura 2-parameter, K2P) [34] from *Miniopterus*, significantly altering the shape of the tree and resulting in difficulties in the estimation of rate heterogeneity parameters. As a consequence, *Myotis* was removed from the analysis in order to aid in the resolution of the tree and to avoid the extensive branch length difficulties reported by recent studies of the phenomenon [35], [36]. The overall topology was unaffected by the removal of the additional individuals and the outgroup.

Molecular clock analyses were conducted using BEAST 1.7.4 [37], [38], incorporating a Yule tree model under a uniform speciation prior. A relaxed uncorrelated lognormal molecular clock [39] was applied using a variable rate of 2.0% sequence evolution per lineage per million years [40]. No further calibration was possible due to the paucity of the fossil record with regard to this group.

All posterior parameter distributions for analysis were checked in Tracer v1.5 [41] for modality and effective sample size (ESS).

Genetic divergence between and within clades were computed as pairwise Kimura 2-parameter distances (K2P) with the software MEGA version 3.1 [42]. The K2P model was chosen to be comparable with previous studies reporting taxonomic inferences on miniopterid bat species based on genetic distances [22], [24], [25], [27], [43].

## Results

Complete or near complete cyt-*b* sequences (1100 to 1140 bp) were obtained for most of the 82 samples sequenced in this study, as well as some critical specimens used in previous taxonomic studies. Exceptions to this were: (1) the paratype of *Miniopterus manavi* (FMNH 5650), a museum skin collected in 1896, and from which 220 bp were obtained; and (2) a tissue sample of FMNH 151718 from which only 710 bp were obtained. All available cyt-*b* sequences, including pre-existing sequences sourced from Genbank, are provided by region and taxon in Table 1. Full specimen details, including Genbank references, are provided in Table S1.

**Table 1:** Number of cyt-*b* sequences by taxon and region included in the present study; with one exception all belong to the genus *Miniopterus*.

Region	Species/clade	Number of sequences
Madagascar	*M. sororculus*	17
	X3	1
	*M. mahafaliensis*	19
	*M. griveaudi*	47
	*M. brachytragos*	12
	*M. manavi*	5
	*M. petersoni*	11
	P6	10
	P7	2
	P5	3
	P4	5
	P3	2
	*M. egeri*	13
	*M. majori*	38
	*M. griffithsi*	6
	*M. gleni*	28
	*M. aelleni* A	15
	*M. aelleni* B	24
Africa	*M. minor*	9
	*M. fraterculus*	10
	*M. natalensis*	14
	*M. newtoni*	4
Europe	*M. schreibersii*	10
Australasia/Asia	*M. australis*	1
	*M. macrocneme*	1
	*M. oceanensis bassanii*	1
	*M. oceanensis orianae*	1
	*M. blepotis*	1
	*M. fuliginosus*	3
	*Myotis ricketti*	1

Full details including Genbank numbers and literature references are included in Table S1.

**Figure 2:**
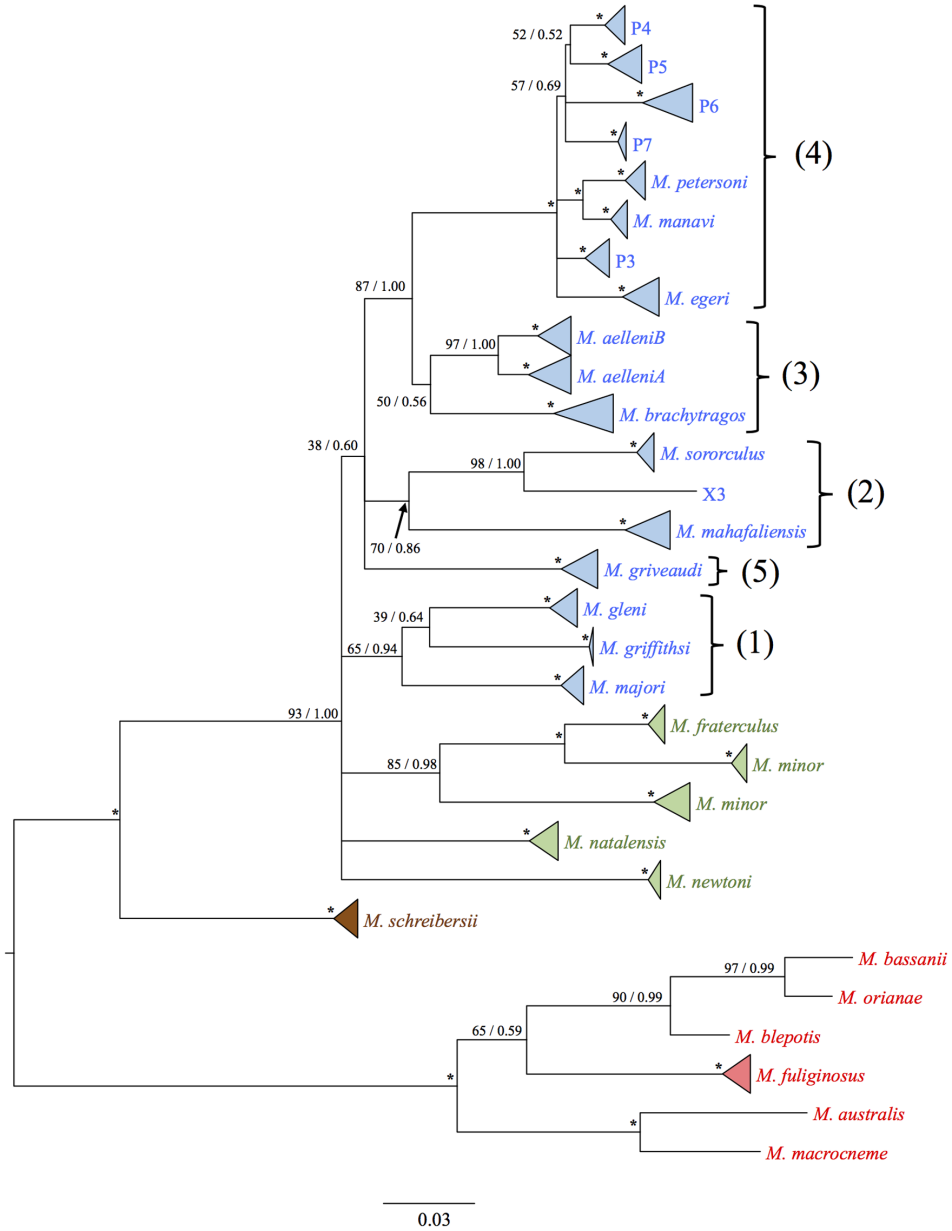
Bayesian majority consensus tree based on cvt-*b* sequence data and according to a HKY + G nucleotide substitution model. The first number at each node represents bootstrap support according to the Maximum Likelihood analysis; the second represents Bayesian posterior probability. An asterisk (*) at a node indicates full support from both analyses, i.e. 100/1.00. Where clades contain more than a single individual, these have been collapsed into triangles. Colour coding refers to the origin of the species, as follows: Blue  =  Madagascar; Green  =  Africa; Brown  =  Europe; Red  =  Asia and Australasia. Large bold numbers beside lineages indicate the five primary lineages referred to in the text.

The initial ML and Bayesian analyses recovered the 264 specimens of Malagasy *Minioptserus* included in this study, as 18 clades (Figure S1). The X3 clade is represented by a single individual. The two to four most divergent haplotypes in each clade were then used for more extensive Bayesian, BEAST and ML analyses. The resulting ML and Bayesian phylogenetic trees produced broadly similar tree topologies, which recovered the reduced set of 54 of Malagasy *Miniopterus* included in this study as 18 clades (Figure 2). Each of these clades received 100% bootstrap (ML) and 1.00 posterior probability (Bayesian) support.

The 18 clades further clustered into five primary lineages. One of these, *M*. *griveaudi*, encompassed a single species. The remaining four sub-clades were comprised as follows: 1) the three taxa *M*. *gleni*, *M*. *griffithsi* and *M*. *majori*, supported with 0.94 posterior probability; 2) the three taxa *M*. *sororculus*, X3 and *M*. *mahafaliensis*, supported with 0.86 posterior probability; 3) the three taxa *M*. *aelleni*A, *M*. *aelleni*B and *M*. *brachytragos* with a lower support of 0.56; and 4) a sub-clade including *M*. *petersoni*, *M*. *manavi*, *M*. *egeri* and the genetically distinct populations referred to herein as P3, P4, P5, P6 and P7, with an overall support of 1.00. Sister relationships between 1) *M*. *petersoni* and *M*. *manavi*; 2) *M*. *aelleni*A and *M*. *aelleni*B; 3) and *M*. *sororculus* and X3 were all supported within their respective lineages at 1.00. The African taxa including *M*. *fraterculus* and *M*. *minor*, as well as the Malagasy *M*. *gleni/M*. *griffithsi/M*. *majori* lineage, formed a polytomy with *M*. *natalensis*, *M*. *newtoni* and the remainder of the Malagasy species. This is most likely due to the effect of a rapid radiation combined with the fast rate of evolution and fixation of the mitochondrial genome.

**Figure 3:**
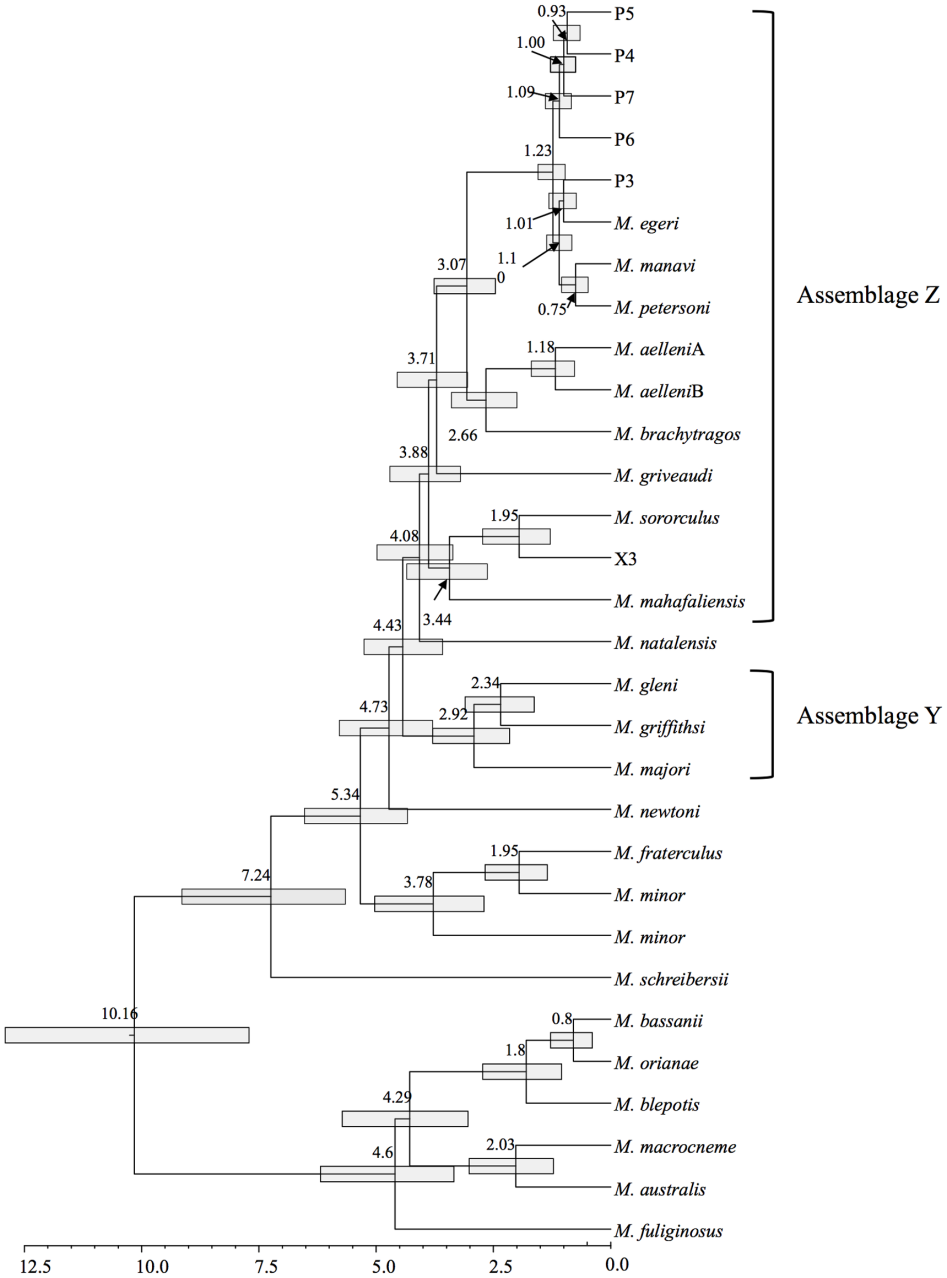
BEAST molecular clock analysis of representative cvt-*b* sequences, incorporating a HKY + G nucleotide substitution model and a Yule model of speciation. Molecular evolutionary rates were calibrated at 2% per million years under a relaxed lognormal clock. Numbers at nodes refer to the age of the node in millions of years (my); the scale bar indicates branch length in my. Grey bars represent 95% highest posterior distributions around node age estimates. Assemblages Y and Z are indicated as noted in the text.

The Beast analysis (Figure 3) revealed that the initial divergence within the Malagasy *Miniopterus* radiation took place at about 4.5 million years ago (Mya). This is, therefore, the minimum date proposed for colonization of Madagascar by *Miniopterus*. Most of the species level divergences are recorded from the period between 4 and 2 Mya. A second set of diversifications took place 1.25 to 0.75 Mya, although uncertainty around these estimates allows some minor overlap between these two diversification periods.

Levels of DNA divergences between the recognized species of Malagasy *Miniopterus* ranged from 12.9% to 2.5% Kimura 2-parameter (K2P) (Table 1). Levels of within species variation were less than 1.8% K2P.

## Discussion

### Origins of Malagasy *Miniopterus*

The DNA phylogenetic analyses recovered Malagasy and African *Miniopterus* spp. as a monophyletic clade relative to Asian and Australasian taxa (Figure 2). Although some Malagasy bird species, which have similar capacity to bats for flight dispersal, appear to have originated through colonization events from Asia and Australasia across the Indian Ocean [44], [45], the cyt-*b* data of the present study clearly supports an African origin for Malagasy *Miniopterus*. With the recent taxonomic revision of Malagasy members of the tribe Emballonurni [46], the only remaining bat genus occurring on Madagascar that is demonstrably Asiatic in origin is the large and strong-flying *Pteropus*.

The available mtDNA data did not resolve conclusively whether there were one or multiple colonization events from Africa into Madagascar. The 18 clades identified among the Malagasy *Miniopterus* clustered into five primary lineages, but these were not recovered as a monophyletic assemblage, relative to African taxa (Figures 2 and 3). The prevailing winds in the nearly 400 km stretch of water separating Madagascar from Africa are westerly since the early Cenozoic [47] and well before the evolution of *Miniopterus*, indicating that colonizations in an easterly direction (i.e. Africa to Madagascar) would be against the prevailing winds. Under this scenario and based on extrapolation from a variety of flying and terrestrial vertebrates [10]–[13], [48], it is unlikely that the five identified Malagasy lineages of *Miniopterus* would each represent a separate colonization event.

Although the five primary lineages were not recovered as a monophyletic group, two major assemblages were identifiable among the Malagasy species: (1) *M*. *gleni*, *M*. *griffithsi* and *M*. *majori*; and (2) the remaining taxa. These two assemblages, referred to as Y and Z (Figure 3), diverged approximately 4.5 Myr ago. They represent either two separate colonization events or a single event that was followed by early divergence. Current data does not allow for the rejection of either of these hypotheses.

The BEAST analysis (Figure 3) also indicated that the five primary Malagasy lineages arose over a period of 3 to 4 Myr ago. There was a pulse of diversification in both assemblages Y and Z around 2 to 3 Myr ago and a further one in assemblage Z within the last million years. As Madagascar's fossil record has a major gap from the Late Cretaceous to Late Quaternary, little is known about the existing habitats and climatic regime on the island during the inferred Pliocene-Pleistocene period of *Miniopterus* diversification.

A caveat to the preceding discussion is that only a single MtDNA gene (cyt-*b*) was analyzed in this study. Although this gene has proven highly useful in identifying cryptic taxonomic diversity in *Miniopterus* [21]–[24] it is clear that further mtDNA augmented with nuclear sequence data is required to better resolve the early radiation and colonization history of the genus in Madagascar. The clarification of the number of taxa as discussed further below will set the framework for more detailed sequencing analyses.

### Diversification of Malagasy *Miniopterus*

The various phylogenetic analyses (ML, Bayesian) all recovered the same 18 clades (Figure 2) of Malagasy *Miniopterus*. Eleven of these correspond directly with currently recognized species [18], [19], [24], [27], [49]–[51]. The other seven clades may represent additional species level diversity, but in certain cases other markers will be needed to resolve relationships.

In terms of DNA distances, the lowest recorded level between recognized sister species involved *M*. *petersoni* and *M*. *manavi*, where DNA distances ranged from 2.5% to 3.3% across the different haplotypes (Table 2). Distances between these two species and *M*. *egeri* ranged from 3.7% to 5.3%, while those involving comparisons between *M*. *majori*, *M*. *gleni* and *M*. *griffithsi* were higher still, ranging from 7.3% to 8.7% (Table 2). These relationships provide context for assessing the level of cyt-*b* differentiation recorded between *M*. *sororculus* and taxon X3. Although recovered as sister taxa, the two differed by a DNA distance of 7.2%, which is consistent with species level differentiation. As currently understood, *M*. *sororculus* is restricted to the central and southern portions of the Central Highlands and the single known individual referred to the X3 clade is from the foothills (810 m) of the central portion of the Central Highlands (Table 3). These two clades are not known to occur in sympatry and most likely represent an example of allopatric speciation that occurred 2 Myr ago based on the BEAST analysis (Figure 3). If this relationship is supported with additional specimens and sequence data, a taxonomic diagnosis for X3 will be required.

**Table 2:** mtDNA distances between Malagasy taxa belonging to the genus *Miniopterus* based on Kimura distances [34].

Comparison between taxa	Distance range %	Comparison within taxa	Maximum distance %
*majori* vs *griffithsi*	7.6 – 8.5	*majori*	1.1
*majori* vs *gleni*	8.3 – 8.7	*griffithsi*	0.3
*griffithsi* vs *gleni*	7.3 – 7.9	*gleni*	1.0
*petersoni* vs *manavi*	2.5 – 3.3	*manavi*	1.8
*manavi-petersoni* vs P complex	3.2 – 5.3	*petersoni*	1.1
*manavi-petersoni* vs *egeri*	3.7 – 5.3	*egeri*	1.7
*egeri* vs P7	4.0 – 5.3	P7	0.3
*egeri* vs P6	4.6 – 6.9	P6	1.6
*egeri* vs P5	3.8 – 5.3	P5	1.6
*egeri* vs P4	3.3 – 4.5	P4	0.4
*egeri* vs P3	2.9 – 4.1	P3	0.7
within the P complex	2.5 – 6.9		
*aelleni*A vs *aelleni*B	3.1 – 3.6	*aelleni*A	1.7
		*aelleni*B	1.5
*sororculus* vs X3	7.2	*sororculus*	0.5

**Table 3:** Summary of different size and life-history parameters of Malagasy (M) and Comorian (C) *Miniopterus* spp. [19], [52].

Taxon	Body Size	Elevation (m)	Distribution	Habitat
*sororculus*	MB	950–2200	C, S	mhf, oh, sbf
X3	SB	810	E	lhf
*mahafaliensis*	SB	0–950	C, S, W	ddf, oh, sbf
*griveaudi* (M)	SB	0–600	N, W	ddf
*griveaudi* (C)	SB	0–900	Grande Comore, Anjouan	lhf, oh
*majori*	MB	0–1550	C, N, S	lhf, mhf, oh
*griffithsi*	LB	25–110	S	sbf
*gleni*	LB	0–1200	E, N, W, S	ddf, lhf, mhf, oh, sbf
*brachytragos*	SB	0–600	E, N, W	ddf, lhf
*aelleni*A (M)	SB	40–500	N, W	ddf
*aelleni*A (C)	SB	220–700	Anjouan	lhf
*aelleni*B	SB	810–1340	N, C	lhf, mhf
*manavi*	SB	900–1500	E, C	mhf, oh
*petersoni*	MB	10–550	S, E	lhf, oh
*egeri*	SB	0–550	N, E	lhf
P3	SB	810–1340	N, C	lhf, mhf
P4	SB	800–875	E	lhf
P5	SB	50–1340	E, C	lhf, mhf
P6	SB	60–1425	C, W	ddf, mhf, oh
P7	SB	1340–1425	C	mhf, oh

Body size: based on mean forearm length (FA), and animals are designated as small-bodied (SB), medium-bodied (MB) and large-bodied (LB); Distribution: E  =  east, N  =  north, W =  west, S  =  south, C  =  central and for the Comoros the name of the island is presented; Habitat: lhf  =  lowland humid forest, mhf  =  montane humid forest, oh  =  open habitat (anthropogenic), ddf  =  dry deciduous forest, sbf  =  spiny bush forest.

The *M*. *aelleni*A and *M*. *aelleni*B clades were less differentiated with distances ranging from 3.1% to 3.6% across the different haplotypes. Nevertheless, this is comparable to levels recorded between closely related and morphologically distinct species such as *M*. *petersoni* and *M*. *manavi*, as well as between these two species and *M*. *egeri* (Table 2). In this context, the two *M*. *aelleni* clades are best treated as separate species. The type series of *M*. *aelleni* includes individuals from the *M*. *aelleni*A clade [27]. Consequently, a taxonomic diagnosis for *M*. *aelleni*B is required. The *M*. *aelleni*A clade includes individuals taken in dry deciduous forests, three of the four being from Ankarana, while those in the *M*. *aelleni*B clade are from humid forest formations, three being from Montagne d’Ambre (Table 3). The sites of Ankarana and Montagne d’Ambre are in close geographical proximity (about 40 km) and share numerous faunistic elements [52].

The five taxa assigned to the P-group were genetically differentiated at levels comparable to those separating *M*. *manavi* and *M*. *petersoni* (Table 2). However, additional markers, specifically based on nuclear DNA, are needed to differentiate between a single genetically variable taxon or several distinct species. It is important to note that the clade assigned to *M*. *manavi* is based on sequence data from a paratype of this species [24]. The most closely related forms were P3, P4, and P7 with genetic distances between them ranging from 2.5% to 3.3% (Table 2). Within each taxon, haplotypes differed by 0.3% to 0.7%. Genetic distances involving comparisons with P6 ranged from 3.2% to 5.3%, while those involving P5 ranged from 2.7% to 5.1%. Within both P5 and P6, haplotype variation did not exceed 1.6%. Distances between *M*. *egeri* and the various P taxa ranged from 2.9% to 6.9%.

The geographical distribution of the P clades provides further insights into the patterns of taxonomic and genetic diversity, although the following conclusions will need to be verified with further field data. P3 is largely restricted to the central portions of the Central Highlands, notably at Ambohitantely, where it shares a day roost with members of the *M*. *manavi*, P7, *M*. *aelleni*B, and P5 clades. It also occurs at sites in the Northern Highlands [53], specifically the Anjanaharibe-Sud and Marojejy Massifs. The form P4 is restricted to a relatively well-defined region at the foot of the Central Highlands (three individuals are from Midongy Sud and the fourth individual is from Andringitra) and all taken between 800 and 875 m. The single known individual of the X3 clade was also collected at Andringitra at 810 m. The form P5 shows a relatively broad distribution, with two individuals taken at Ambohitantely in the Central Highlands and at same day roost site as *M*. *manavi*, *M*. *aelleni*B, P7 and P3, as well as at 50 m elevation in the eastern lowlands. P7 also occurs in the Central Highlands (Ambohitantely) and Fanadana co-occurring with *M*. *manavi*, P6 and *M*. *majori*. The form P6 is widespread and includes individuals from the central western site of Namoroka, the northwestern offshore island of Nosy Be, and Fanandana.

The fact that each P clade was recovered as monophyletic, combined with the levels of genetic divergences between clades, and the co-occurrence at Ambohitantely and Fanandana of several of the P3 to P7 clades, requires further comment. One interpretation is that the P clades represent distinct but closely related cryptic species. The previous identification of additional species diversity in the *M*. *manavi* complex (e.g. *M*. *petersoni*, *M*. *egeri*) has been defined by both DNA and morphological evidence, such as tragus shape [19], [27], [49]. An examination of the tragi of animals from Ambohitantely belonging to clades P5 and P7 did not disclose noticeable differences in tragus structure. It may be that the P forms are incipient species and although genetic separation has occurred, obvious morphological differences have not yet evolved.

The P clade may comprise cases of incomplete lineage sorting of the mitochondrial genome, introgression and hybridization of closely related taxa or possibly a combination of these processes [54], [55]. The source taxon, however, is not clear from the available data: none of the P haplotypes were associated with any named species. Further analysis of this complex will require multiple nuclear markers in order to resolve their taxonomic status and relationship.

One further aspect that may have confounded our analyses of genetic relationships in the *manavi* group, including *M*. *petersoni*, *M*. *egeri* and the P group of clades, is that we used a mitochondrial marker that is only transmitted by females [56]. Hence, our evaluations here are only of the genetic relationships passed on by females, which may not accurately reflect patterns of overall gene flow. In bats, females tend to be notably more philopatric than males [57] and the use of bi-parentally inherited genes might provide further insight into the phylogenetic relationships of these different clades. However, we come to the same question as above: to which species do the P haplotypes align? Clearly, further morphological, ecological and behavioral work is required to understand better the taxonomic status of the five P taxa. At the very least, they may represent a single cryptic species with high levels of haplotype diversity to five currently unrecognized taxa requiring formal description.

### Colonization of the Comoros

It has been demonstrated that two species of *Miniopterus* are shared between Madagascar and the Comoros [58], [59] and include *M*. *griveaudi* and *M*. *aelleni* [27]. Using mtDNA and microsatellites, it was concluded that *M*. *griveaudi* colonized the Comoros from Madagascar some 180 000 years ago [58]. Although this suggests a similar colonization history for *M*. *aelleni*, DNA differentiation was not compared between Malagasy and Comorian populations of the latter because of low sample sizes [58].

Herein, our study reveals that individuals of *M*. *aelleni* from Comoros are nested within the *M*. *aelleni*A clade (Figure 2). This indicates a recent colonization of the Comoros from a member of this apparent species complex. As the principal four islands of the Comoros have never been connected to other landmasses, dispersal would have been over-water in a westerly direction and following the prevailing winds.

Although *M*. *griveaudi* and *M*. *aelleni*A are not phylogenetically closely related, they are similar in their habitats, distribution and morphology (Table 3). Both taxa occur in dry deciduous forests in Madagascar. The ability to colonize islands in these two taxa is likely linked to similar ecological parameters. Interestingly, the populations in the Comoros of both *M*. *griveaudi* and *M*. *aelleni*A occur in lowland humid forest, the former also occurring in open habitat (anthropogenic).

### Patterns of ecological diversification and speciation in Madagascar

All of the individuals in the P3 to P7 clades occur in the eastern humid forests at high (Central Highlands) to low (Sahafina) elevations (Table 3), with only a few exceptions: P6 includes two individuals from the dry deciduous forest site of Namoroka and one individual from the transitional dry deciduous/humid forest Sambirano formation of Nosy Be. Thus, most of the genetic diversity has been generated in the east. Based on the Beast analysis for extant species, the differentiation of the Malagasy *Miniopterus* radiation commenced about 4.5 Myr ago, with two periods of cladogenesis: 4–2 Myr and 0.75–1.25 Myr. These are periods for which little hard data exist for environmental conditions on the island and an interpretation of the ecological and evolutionary forces that gave rise to this differentiation is not evident. However, given the high level of syntopic occurrence of members of the P group, specifically at Ambohitantely and Fanandana, it is possible that past fragmentation of populations followed by range expansion are related, for example, to the cyclic climate changes of the Late Pleistocene/Early Holocene [60], [61], particularly in more montane zones, such as the Central Highlands, where Ambohitantely and Fandanana are found.

Using measures of species diversity of *Miniopterus* on Madagascar and the Comoros, the number of recognized species has gone from four in 1995 [17] to eleven currently recognized species [18], [19]. When the current genetic data are analyzed together, there are indications that, at least from the perspective of phylogenetic species, something approaching 18 taxa occur on Madagascar. Similar studies of continental African *Miniopterus* reveal that levels of species richness are higher than current estimates would indicate [62] and that, for example *M. minor* is paraphyletic (Figures 2 and 3). We suspect the same pattern will be found in other portions of the Old World range of this genus. Before the recent wave of molecular studies of members of this genus, something approaching 20 species were recognized across its Old World distribution [63]. If the patterns of cryptic species richness on Madagascar hold for other areas, it is conceivable that over a hundred taxa comprise this genus, making it an example of one of the most successful adaptive radiations amongst bats.

## Supporting Information

Figure S1 **Maximum Likelihood (ML) tree generated using RaXML Blackbox (see main text for citation) and derived from all available** ***cyt*** **-b sequences, corroborated with Bayesian analysis using MrBayes 3.2 (see main text for citation).**
(DOCX)

Table S1 **Specimen data for individuals included in the study.**
(DOCX)
