# Supplementary material for: Insights into the Evolution of a Cryptic Radiation of Bats: Dispersal and Ecological Radiation of Malagasy Miniopterus (Chiroptera: Miniopteridae)
Source: PLoS One. 2014 Mar 18;9(3):e92440. doi: 10.1371/journal.pone.0092440 (PMC3958536; doi:10.1371/journal.pone.0092440)
Supplement: Figure S1 — Maximum Likelihood (ML) tree generated using RaXML Blackbox (see main text for citation) and derived from all available cyt -b sequences, corroborated with Bayesian analysis using MrBayes 3.2 (see main text for citation). (DOCX) [file pone.0092440.s001.docx]

**Supplementary Figure S1.** Maximum Likelihood (ML) tree generated using RaXML Blackbox (see main text for citation) and derived from all available *cyt*-b sequences, corroborated with Bayesian analysis using MrBayes 3.2 (see main text for citation). The native rate heterogeneity correction and substitution models were applied for ML (GTRCAT), while HKY + G was used in MrBayes. Clades containing more than one individual were collapsed and the sample number provided in the label. Numbers at nodes indicate bootstrap support values followed by posterior probabilities. An asterisk (*) indicates full support in both analyses (100 / 1.00), while a hyphen (-) indicates that the node was not recovered or showed support of less than 50 or 0.50.
