# Supplementary material for: Insights into the Evolution of a Cryptic Radiation of Bats: Dispersal and Ecological Radiation of Malagasy Miniopterus (Chiroptera: Miniopteridae)
Source: PLoS One. 2014 Mar 18;9(3):e92440. doi: 10.1371/journal.pone.0092440 (PMC3958536; doi:10.1371/journal.pone.0092440)
Supplement: Table S1 — Specimen data for individuals included in the study. (DOCX) [file pone.0092440.s002.docx]

**Table S1.** Specimen data for individuals included in the study. Sequence sample numbers are provided next to the taxon designation in brackets. These include cytochrome b sequences sourced from GenBank. In some cases, specimen number and locality data are limited; an asterisk (*) indicates that more precise locality data was not available for the individual. A double asterisk (**) indicates individuals currently identified as subspecies of *Miniopterus schreibersii*; however, these are currently under taxonomic revision and should be considered to possess species status (in prep.). Note: some individuals share an identical GenBank reference although they possess a unique specimen number. All these individuals have been sequenced; however, where they exhibited identical cytochrome b haplotypes, they were submitted under the same reference. All new sequences acquired for this study can be found under GenBank numbers KJ095124-KJ095198.

Reference numbers indicate the study from which sequences are sourced: a full list of these references is provided below:

**SEQUENCE REFERENCES**

1. Present study
2. Cardinal BR, Christidis L (2000) Mitochondrial DNA and morphology reveal three geographically distinct lineages of the large bentwing bat (*Miniopterus schreibersii*) in Australia. Aust J Zool 48: 1–19.
3. Garcia-Mudarra JL, Ibanez C, Juste J (2209) The Straits of Gibraltar: barrier to Ibero-Moroccan bat diversity? Biol. J. Linn. Soc. Lond. 96: 434-450.
4. Goodman SM, Ryan KE, Maminirina CP, Fahr J, Christidis L, et al. (2007) The specific status of populations on Madagascar referred to *Miniopterus fraterculus* (Chiroptera: Vespertilionidae), with description of a new species. J Mam: 1216–1229.
5. Goodman SM, Bradman HM, Maminirina CP, Ryan KE, Christidis L, et al. (2008) A new species of *Miniopterus* (Chiroptera: Miniopteridae) from lowland southeastern Madagascar. Mam Biol 73: 199–213.
6. Goodman SM, Maminirina CP, Bradman HM, Christidis L, Appleton B (2009a) The use of molecular phylogenetic and morphological tools to identify cryptic and paraphyletic species: examples from the diminutive long-fingered bats (Chiroptera: Miniopteridae: *Miniopterus*) on Madagascar. Am Mus Novit 3669: 1–34.
7. Goodman SM, Maminirina CP, Weyeneth N, Bradman HM, Christidis L, et al. (2009b) The use of molecular and morphological characters to resolve the taxonomic identity of cryptic species: the case of *Miniopterus manavi* (Chiroptera, Miniopteridae). Zool Scripta 38: 339–363.
8. Goodman SM, Maminirina CP, Bradman HM, Christidis L, Appleton B (2010) Patterns of morphological and genetic variation in the endemic Malagasy bat *Miniopterus gleni* (Chiroptera: Miniopteridae), with the description of a new species, *M. griffithsi*. J Zool Syst Evol Res 48: 75–86.
9. Goodman SM, Ramasindrazana B, Maminirina CP, Schoeman MC, Appleton B (2011) Morphological, bioacoustical, and genetic variation in *Miniopterus* bats from eastern Madagascar, with the description of a new species. Zootaxa 2880: 1–19.
10. Ibanez C, Garcia-Mudarra JL, Ruedi M, Stadelmann B, Juste J (2006) The Iberian contribution to cryptic diversity in European bats. Acta Chiropterol 8: 277-297.
11. Juste J, Ferrández A, Fa JE, Masefield W, Ibáyez C (2007) Taxonomy of little bent-winged bats (*Miniopterus*, Miniopteridae) from the African islands of Sao Tomé, Grand Comoro and Madagascar, based on mtDNA. Acta Chiropterol 9: 27–37.
12. Maminirina CP, Appleton B, Bradman HM, Christidis L, Goodman SM (2009) Variations géographique et moléculaire chez *Miniopterus majori* (Chiroptera : Miniopteridae) de Madagascar. Malagasy Nature 2: 127-143.
13. Miller-Butterworth CM, Eick G, Jacobs DS, Schoeman MC, Harley EH (2005) Genetic and phenotypic differences between South African long-fingered bats, with a global miniopterine phylogeny. J Mam 86: 1121-1135.
14. Niu H, Ma H (2007) Molecular study on systematics and population of *Miniopterus schreibersii* (Chiroptera: Vespertilionidae). Direct submission to GenBank.
15. Ramasindrazana B, Goodman SM, Schoeman MC, Appleton B (2011) Identification of cryptic species of Miniopterus bats (Chiroptera: Miniopteridae) from Madagascar and the Comoros using bioacoustics overlaid on molecular genetic and morphological characters. Biol J Linn Soc 104: 284-302.
16. Ruedi M, Mayer F (2001) Molecular systematics of bats of the genus *Myotis* (Vespertilionidae) suggests deterministic ecomorphological convergences. Mol. Phylogenet Evol 21: 436-448.
17. Sakai T, Kikkawa Y, Tsuchiya K, Harada M, Kanoe M, Yoshiyuki M, Yonekawa H (2003) Molecular phylogeny of Japanese Rhinolophidae based on variations in the complete sequence of the mitochondrial cytochrome b gene. Genes Genet Syst 78: 179-189.
18. Stadelmann B, Jacobs DS, Schoeman C, Ruedi M (2004) Phylogeny of African Myotis bats (Chiroptera, Vespertilionidae) inferred from cytochrome b sequences. Acta Chiropterol 6: 177-192.
19. Weyeneth N, Goodman SM, Stanley WT, Ruedi, M (2008) The biogeography of *Miniopterus* bats (Chiroptera: Miniopteridae) from the Comoro Archipelago inferred from mitochondrial DNA. Mol Ecol 17: 5205–5219.
20. Weyeneth N, Goodman SM, Appleton B, Wood R, Ruedi M (2011) Wings or winds: inferring bat migration in a stepping-stone archipelago. J Evol Biol 24: 1298–1306.

| **Species/clade** | **Specimen label** | **Locality** | **GenBank No.** | **Reference** |
| --- | --- | --- | --- | --- |
| *M. sororculus*  (17) | USNM 577120 | Ranopiso | KJ095194 | 1 |
|  | USNM 577121 | Ranopiso | KJ015195 | 1 |
|  | FMNH 184467 | Ihosy | KJ095196 | 1 |
|  | FMNH 177257 | Ambatofinandraha | DQ899770 | 4 |
|  | FMNH 177258 | Ambatofinandraha | DQ899772 | 4 |
|  | FMNH 177264 | Ambatofinandraha | DQ899773 | 4 |
|  | FMNH 177262 | Antsampandrano | DQ899774 | 4 |
|  | FMNH 177259 | Antsampandrano | DQ899771 | 4 |
|  | FMNH 177267 | Antsampandrano | HQ619937 | 9 |
|  | FMNH 209180 | Fandanana | JF440282 | 15 |
|  | UADBA 43201 | Fandanana | JF440283 | 15 |
|  | UADBA 43202 | Fandanana | JF440284 | 15 |
|  | FMNH 209181 | Fandanana | JF440285 | 15 |
|  | FMNH 209182 | Fandanana | JF440286 | 15 |
|  | FMNH 209183 | Fandanana | HQ619938 | 9 |
|  | FMNH 209184 | Fandanana | KJ095197 | 1 |
|  | FMNH 209185 | Fandanana | DQ899774 | 1 |
| X3 (1) | FMNH 151718 | Andringitra | KJ095198 | 1 |
| *M. mahafaliensis*  (19) | FMNH 184470 | Ihosy | FJ383166 | 6 |
|  | FMNH 173197 | Tsimanampetsotsa | FJ383160 | 6 |
|  | FMNH 172919 | Sarodrano | FJ383163 | 6 |
|  | FMNH 172930 | St Augustin | FJ383168 | 6 |
|  | FMNH 176096 | Kirindy-Mite | FJ383165 | 6 |
|  | FMNH 176100 | Kirindy-Mite | FJ383161 | 6 |
|  | FMNH 176101 | Kirindy-Mite | FJ383167 | 6 |
|  | FMNH 176103 | Kirindy-Mite | FJ383164 | 6 |
|  | FMNH 176104 | Kirindy-Mite | FJ383162 | 6 |
|  | FMNH 175991 | Isalo | FJ383166 | 6 |
|  | UADBA SMG 16472 | Tsimanampetsotsa | KJ095159 | 1 |
|  | FMNH 209275 | Tsimanampetsotsa | KJ095160 | 1 |
|  | FMNH 209276 | Tsimanampetsotsa | JF440264 | 15 |
|  | FMNH 209277 | Tsimanampetsotsa | JF440265 | 15 |
|  | FMNH 209278 | Tsimanampetsotsa | JF440266 | 15 |
|  | UADBA 43221 | Itampolo | JF440267 | 15 |
|  | FMNH 209279 | Itampolo | JF440268 | 15 |
|  | FMNH 209281 | Itampolo | JF440269 | 15 |
|  | UADBA SMG 16471 | Tsimanampetsotsa | KJ095158 | 1 |

| **Species/clade** | **Specimen label** | **Locality** | **GenBank No.** | **Reference** |
| --- | --- | --- | --- | --- |
| *M. griveaudi*  (46) | FMNH 179377 | Andavakoera | FJ383136 | 7 |
|  | FMNH 169681 | Bemaraha | DQ899767 | 4 |
|  | FMNH 173274 | Ankarana | DQ899766 | 4 |
|  | FMNH 172817 | Bemaraha | FJ383144 | 7 |
|  | FMNH 179232 | Anjohikely | FJ232793 | 19 |
|  | FMNH 179233 | Anjohikely | FJ383145 | 7 |
|  | FMNH 179234 | Anjohikely | FJ383143 | 7 |
|  | FMNH 194260 | Union des Comores | FJ383139 | 7 |
|  | FMNH 194263 | Union des Comores | FJ232857 | 19 |
|  | FMNH 194273 | Union des Comores | FJ383140 | 7 |
|  | FMNH 194277 | Union des Comores | FJ232796 | 18 |
|  | FMNH 194289 | Union des Comores | FJ383137 | 7 |
|  | FMNH 194390 | Union des Comores | FJ383141 | 7 |
|  | FMNH 194394 | Union des Comores | FJ232799 | 19 |
|  | FMNH 194405 | Union des Comores | FJ232797 | 20 |
|  | FMNH 194417 | Union des Comores | FJ232794 | 20 |
|  | FMNH 194426 | Union des Comores | FJ232814 | 19 |
|  | FMNH 194433 | Union des Comores | FJ383138 | 7 |
|  | FMNH 173101 | Ankarana | FJ383136 | 7 |
|  | FMNH 175839 | Namoroka | FJ383142 | 7 |
|  | UADBA 43190 | Belobaka | JF440242 | 15 |
|  | UADBA 43191 | Belobaka | JF440243 | 15 |
|  | UADBA 43192 | Belobaka | JF440244 | 15 |
|  | UADBA 43193 | Belobaka | JF440245 | 15 |
|  | UADBA 43194 | Belobaka | JF440246 | 15 |
|  | UADBA 43195 | Belobaka | Kj095197 | 1 |
|  | UADBA 43196 | Belobaka | JF440247 | 15 |
|  | FMNH 209256 | Belobaka | JF440248 | 15 |
|  | FMNH 209257 | Belobaka | JF440249 | 15 |
|  | FMNH 209247 | Ambinda | JF440250 | 15 |
|  |  | Union des Comores | EF363519 | 11 |
|  |  | Union des Comores | EF363518 | 11 |
|  |  | Union des Comores | EF363517 | 11 |
|  | FMNH 220045 | Union des Comores | JF440260 | 15 |
|  | FMNH 220052 | Union des Comores | JF440262 | 15 |
|  | FMNH 220062 | Union des Comores | JF440258 | 15 |
|  | UADBA 48131 | Ambinda | KJ095155 | 1 |
|  | FMNH 209248 | Ambinda | JF440251 | 15 |
|  | UADBA SMG 16543 | Ambinda | JF440252 | 15 |
|  | FMNH 209246 | Ambinda | KJ095156 | 1 |
|  | FMNH 220049 | Union des Comores | JF440263 | 15 |
|  | FMNH 220060 | Union des Comores | JF440257 | 15 |
| **Species/clade** | **Specimen label** | **Locality** | **GenBank No.** | **Reference** |
| *M. griveaudi*  (46) | FMNH 220063 | Union des Comores | JF440259 | 15 |
|  | FMNH 220055 | Union des Comores | JF440256 | 15 |
|  |  | Madagascar* | FJ232802 | 19 |
|  | FMNH 220048 | Union des Comores | JF440261 | 15 |
| *M. brachytragos*  (12) | FMNH 175840 | Namoroka | FJ383152 | 6 |
|  | FMNH 175846 | Namoroka | FJ383151 | 6 |
|  | FMNH 175850 | Namoroka | FJ383158 | 6 |
|  | FMNH 175852 | Namoroka | FJ383154 | 6 |
|  | FMNH 175856 | Namoroka | FJ383157 | 6 |
|  | FMNH 175870 | Namoroka | FJ383156 | 6 |
|  | FMNH 175871 | Namoroka | FJ383155 | 6 |
|  | FMNH 175873 | Namoroka | FJ383150 | 6 |
|  | FMNH 175875 | Namoroka | FJ383153 | 6 |
|  | FMNH 175877 | Namoroka | FJ383154 | 6 |
|  | FMNH 188651 | Nosy Komba | FJ383159 | 6 |
|  | FMNH 202523 | Ambanizana | FJ383149 | 6 |
| *M. manavi*  (4) | FMNH 5650 | Vinanitelo | FJ383128 | 7 |
|  | FMNH 187662 | Ranomafana | FJ383129 | 7 |
|  | FMNH 194074 | Fandanana | FJ383130 | 7 |
|  | UADBA 43171 | Ambohitantely | HQ619934 | 9 |
| *M. petersoni*  (11) | USNM 577096 | Manantantely | FJ383131 | 7 |
|  | USNM 577097 | Manantantely | EU091258 | 5 |
|  | USNM 577098 | Manantantely | FJ383131 | 5 |
|  | USNM 577102 | Nahampoana | KJ095192 | 5 |
|  | UADBA 47312 | Nahampoana | EU091255 | 5 |
|  | USNM 577104 | Nahampoana | EU091257 | 5 |
|  | USNM 577105 | Nahampoana | FJ383131 | 5 |
|  | USNM 577106 | Nahampoana | KJ095193 | 5 |
|  | FMNH 194136 | Manantantely | EU091257 | 5 |
|  | USNM 577127 | Manantenina | EU091254 | 5 |
|  | FMNH 209186 | Manantantely | EU091257 | 5 |
| P6  (10) | FMNH 175843 | Namoroka | KJ095188 | 1 |
|  | FMNH 175845 | Namoroka | KJ095189 | 1 |
|  | FMNH 209178 | Fandanana | JF440280 | 15 |
|  | FMNH 187771 | Nosy Be | KJ095186 | 1 |
|  | FMNH 187772 | Nosy Be | KJ095187 | 1 |
|  | FMNH 194553 | Kandreho | KJ095190 | 1 |
|  | UADBA 43183 | Ambohitantely | KJ095185 | 1 |
|  | UADBA 43181 | Ambohitantely | KJ095184 | 1 |
|  | UADBA 43177 | Ambohitantely | KJ095183 | 1 |
| P7  (2) | FMNH 194168 | Ambohitantely | KJ095191 | 1 |
|  | FMNH 209179 | Fandanana | JF440281 | 15 |
| **Species/clade** | **Specimen label** | **Locality** | **GenBank No.** | **Reference** |
| P5  (3) | FMNH 194162 | Ambohitantely | KJ095182 | 1 |
|  | FMNH 194164 | Ambohitantely | KJ095180 | 1 |
|  | FMNH 209162 | Sahafina | KJ095181 | 1 |
| P4  (5) | FMNH 178616 | Befotaka | KJ095176 | 1 |
|  | FMNH 178618 | Befotaka | KJ095177 | 1 |
|  | FMNH 178622 | Befotaka | KJ095178 | 1 |
|  | FMNH 178623 | Befotaka | KJ095179 | 1 |
|  | FMNH 151714 | Andringitra | KJ095175 | 1 |
| P3  (2) | FMNH 167450 | Anjanaharibe-sud | FJ383132 | 7 |
|  | FMNH 172602 | Marojejy | FJ383133 | 7 |
|  | UADBA 43179 | Ambohitantely | KJ095174 | 1 |
| *M. egeri*  (13) | USNM 448938 | Kianjavato | HQ619940 | 9 |
|  | USNM 449202 | Vatovavy | HQ619941 | 9 |
|  | FMNH 187663 | Kianjavato | HQ619947 | 9 |
|  | FMNH 187664 | Kianjavato | HQ619946 | 9 |
|  | FMNH 202519 | Farankaraina | HQ619945 | 9 |
|  | FMNH 202520 | Farankaraina | HQ619942 | 9 |
|  | FMNH 202521 | Farankaraina | HQ619944 | 9 |
|  | FMNH 202522 | Ambanizana | HQ619943 | 9 |
|  | FMNH 202474 | Sahafina | HQ619949 | 9 |
|  | FMNH 209160 | Sahafina | HQ619952 | 9 |
|  | FMNH 209161 | Sahafina | HQ619948 | 9 |
|  | FMNH 209168 | Sahafina | HQ619950 | 9 |
|  | FMNH 209159 | Sahafina | HQ619950 | 9 |
| *M. majori*  (38) | FMNH 177275 | Ambatofinandraha | DQ899777 | 4 |
|  | FMNH 177279 | Ambatofinandraha | DQ899778 | 4 |
|  | FMNH 194081 | Fandanana | EU636764 | 12 |
|  | FMNH 194083 | Fandanana | JF440287 | 15 |
|  | FMNH 194085 | Fandanana | EU636769 | 12 |
|  | UADBA 43197 | Fandanana | KJ095162 | 1 |
|  | UADBA 43198 | Fandanana | HQ619955 | 9 |
|  | UADBA 43199 | Fandanana | KJ095163 | 1 |
|  | UADBA 43200 | Fandanana | JF440271 | 9 |
|  | UADBA 43261 | Fandanana | HQ619954 | 9 |
|  | UADBA 43264 | Fandanana | HQ619953 | 9 |
|  | FMNH 209170 | Fandanana | JF440270 | 15 |
|  | UADBA 43259 | Fandanana | JF440270 | 15 |
|  | FMNH 209172 | Fandanana | KJ095164 | 1 |
|  | UADBA 43256 | Fandanana | JF440278 | 15 |
|  | UADBA 43262 | Fandanana | KJ095168 | 1 |
|  | FMNH 209171 | Fandanana | JF440272 | 15 |
|  | FMNH 209177 | Fandanana | KJ095170 | 1 |
|  | UADBA 43253 | Fandanana | KJ095167 | 1 |
|  | UADBA 43253 | Fandanana | JF440273 | 15 |
| **Species/clade** | **Specimen label** | **Locality** | **GenBank No.** | **Reference** |
| *M. majori*  (38) | UADBA 43255 | Fandanana | KJ095165 | 1 |
|  | UADBA 43254 | Fandanana | JF440276 | 15 |
|  | UADBA 43260 | Fandanana | KJ095166 | 1 |
|  | UADBA 43258 | Fandanana | JF440279 | 15 |
|  | FMNH 209173 | Fandanana | JF440274 | 15 |
|  | UADBA 43263 | Fandanana | KJ095169 | 1 |
|  | FMNH 209174 | Fandanana | JF440275 | 15 |
|  | UADBA 43257 | Fandanana | JF440287 | 15 |
|  | FMNH 202518 | Farankaraina | HQ619939 | 9 |
|  | FMNH 177286 | Itremo | DQ899775 | 4 |
|  | FMNH 177287 | Itremo | DQ899779 | 4 |
|  | FMNH 177288 | Itremo | DQ899786 | 4 |
|  | USNM 577075 | Manantantely | KJ095161 | 1 |
|  | FMNH 166135 | Tsinjoarivo | DQ899776 | 4 |
|  | FMNH 209175 | Fandanana | JF440277 | 15 |
|  | JMH 141 | Ankarana | AY614741 | 13 |
|  | JMH 029 | Ranomafana | AY614740 | 13 |
| *M. griffithsi*  (6) | FMNH 184214 | Itampolo | FJ619517 | 8 |
|  | FMNH 184215 | Itampolo | FJ619518 | 8 |
|  | FMNH 184216 | Itampolo | FJ619518 | 15 |
|  | UADBA 43229 | Itampolo | JF440240 | 15 |
|  | FMNH 209274 | Itampolo | JF440241 | 15 |
|  | USNM 577076 | Ranopiso | FJ619518 | 15 |
| *M. gleni*  (28) | FMNH 175832 | Namoroka | DQ899768 | 4 |
|  | FMNH 176309 | Ankarana | DQ899769 | 4 |
|  | FMNH 202524 | Farankaraina | FJ383146 | 7 |
|  | FMNH 202525 | Farankaraina | FJ383146 | 7 |
|  | FMNH 202526 | Ambanizana | KJ095151 | 1 |
|  | FMNH 202528 | Ambanizana | KJ095152 | 1 |
|  | FMNH 202530 | Ambanizana | KJ095144 | 1 |
|  | FMNH 202531 | Ambanizana | FJ383146 | 7 |
|  | FMNH 202533 | Ambanizana | KJ095153 | 1 |
|  | FMNH 202534 | Ambanizana | FJ383147 | 7 |
|  | FMNH 202535 | Ambanizana | KJ095154 | 1 |
|  | FMNH 202536 | Ambanizana | FJ383146 | 7 |
|  | FMNH 202537 | Ambanizana | JF440238 | 15 |
|  | FMNH 202447 | Sarodrano | FJ619516 | 8 |
|  | FMNH 202448 | Sarodrano | JF440236 | 15 |
|  | UADBA 43216 | Sarodrano | KJ095145 | 1 |
|  | UADBA 43217 | Sarodrano | JF440234 | 15 |
|  | UADBA 43218 | Sarodrano | KJ095146 | 1 |
|  | UADBA 43219 | Sarodrano | JF440235 | 15 |
| **Species/clade** | **Specimen label** | **Locality** | **GenBank No.** | **Reference** |
| *M. gleni*  (28) | FMNH 209271 | Sarodrano | JF440236 | 15 |
|  | FMNH 209272 | Sarodrano | KJ095147 | 1 |
|  | FMNH 209273 | Sarodrano | KJ095148 | 1 |
|  | FMNH 209250 | Ambinda | JF440237 | 15 |
|  | FMNH 209251 | Ambinda | JF440238 | 15 |
|  | UADBA 48139 | Ambinda | JF440239 | 15 |
|  | UADBA 48145 | Ambinda | KJ095150 | 1 |
|  | UADBA 48151 | Ambinda | JF440239 | 15 |
|  | FMNH 209252 | Ambinda | KJ095149 | 1 |
| *M. aelleni* A  (15) | FMNH 173070 | Ankarana | FJ232795 | 19 |
|  | FMNH 173089 | Ankarana | FJ232800 | 19 |
|  | FMNH 173091 | Ankarana | KJ095127 | 1 |
|  | FMNH 209243 | Ambinda | JF440219 | 15 |
|  | FMNH 209244 | Ambinda | KJ095124 | 1 |
|  | UADBA 48146 | Ambinda | JF440220 | 15 |
|  | UADBA 48150 | Ambinda | KJ095125 | 1 |
|  | UADBA 43932 | Ankarana | JF440221 | 15 |
|  | UADBA 43933 | Ankarana | JF440223 | 15 |
|  | UADBA 43934 | Ankarana | JF440225 | 15 |
|  | FMNH 173090 | Ankarana | KJ095126 | 1 |
|  | FMNH 175842 | Namoroka | KJ095129 | 1 |
|  | FMNH 175837 | Namoroka | KJ095128 | 1 |
|  | FMNH 194420 | Union des Comores | FJ232801 | 19 |
| *M. aelleni* B  (23) | FMNH 202450 | Montagne d'Ambre | FJ383135 | 7 |
|  | UADBA 43176 | Ambohitantely | KJ095131 | 1 |
|  | UADBA 48635 | Montagne d'Ambre | KJ095142 | 1 |
|  | UADBA 31950 | Montagne d'Ambre | FJ383134 | 7 |
|  | FMNH 202451 | Montagne d'Ambre | KJ095136 | 1 |
|  | FMNH 202449 | Montagne d'Ambre | KJ095135 | 1 |
|  | FMNH 202452 | Montagne d'Ambre | FJ383135 | 7 |
|  | UADBA 31951 | Montagne d'Ambre | FJ383135 | 7 |
|  | FMNH 202453 | Montagne d'Ambre | FJ383148 | 6 |
|  | UADBA 31954 | Montagne d'Ambre | FJ383135 | 7 |
|  | UADBA 48629 | Montagne d'Ambre | KJ095142 | 1 |
|  | UADBA 48634 | Montagne d'Ambre | KJ095141 | 1 |
|  | UADBA 48630 | Montagne d'Ambre | KJ095138 | 1 |
|  | UADBA 48631 | Montagne d'Ambre | KJ095139 | 1 |
|  | UADBA 48632 | Montagne d'Ambre | KJ095140 | 1 |
|  | UADBA 43178 | Ambohitantely | KJ095132 | 1 |
|  | UADBA 43180 | Ambohitantely | KJ095137 | 1 |
|  | UADBA 43175 | Ambohitantely | KJ095130 | 1 |

| **Species/clade** | **Specimen label** | **Locality** | **GenBank No.** | **Reference** |
| --- | --- | --- | --- | --- |
| *M. aelleni* B  (24) | FMNH 194169 | Ambohitantely | KJ095134 | 1 |
|  | UADBA 43172 | Ambohitantely | HQ619935 | 9 |
|  | FMNH 172600 | Marojejy | KJ095133 | 1 |
|  | UADBA 43175 | Ambohitantely | KJ095130 | 1 |
| *M. minor*  (9) | FMNH 198034 | Tanzania | FJ232806 | 19 |
|  | FMNH 198010 | Tanzania | FJ232804 | 19 |
|  | FMNH 198098 | Tanzania | FJ232805 | 19 |
|  | FMNH 198164 | Tanzania | FJ232803 | 19 |
|  | FMNH 205313 | Tanzania | KJ095172 | 1 |
|  | FMNH 205314 | Tanzania | KJ095171 | 1 |
|  | FMNH 205315 | Tanzania | KJ095172 | 1 |
|  | FMNH 205316 | Tanzania | KJ095173 | 1 |
|  | TM41802 | South Africa | AY614737 | 13 |
| *M. fraterculus*  (10) | MR-M988 | South Africa | AJ841975 | 18 |
|  | TM 41638 | South Africa | DQ899761 | 4 |
|  | TM 42120 | South Africa | DQ899760 | 4 |
|  | TM 47722 | Swaziland | EU091246 | 5 |
|  | DSJ1115 | Africa* | AY614750 | 13 |
|  | DSJ179 | South Africa | AY614755 | 13 |
|  | DSJ183 | South Africa | AY614754 | 13 |
|  | DSJ1116 | Africa* | AY614753 | 13 |
|  | DSJ1120 | Africa* | AY614752 | 13 |
|  | DSJ1117 | Africa* | AY614751 | 13 |
| *M. 'natalensis'*  (14) | SKK4 | South Africa | DQ899765 | 4 |
|  | TM 46484 | South Africa | DQ899762 | 4 |
|  | TM 46881 | South Africa | DQ899764 | 4 |
|  | TM 47622 | South Africa | DQ899763 | 4 |
|  | DSJ1030 | South Africa | AY614744 | 13 |
|  | DSJ1078 | South Africa | AY614743 | 13 |
|  | DSJ925 | South Africa | AY614742 | 13 |
|  | DSJ197 | South Africa | AY614748 | 13 |
|  | DSJ790 | South Africa | AY614749 | 13 |
|  | DSJ853 | South Africa | AY614747 | 13 |
|  | DSJ147 | South Africa | AY614745 | 13 |
|  | DSJ860 | South Africa | AY614746 | 13 |
|  | MR-M966 | South Africa | AJ841977 | 13 |
|  | MR-M933 | South Africa | AJ841976 | 13 |
| *M. newtoni*  (4) | EF363520 | Sao Tomé | EF363520 | 11 |
|  | EF363521 | Sao Tomé | EF363521 | 11 |
|  | EF363522 | Sao Tomé | EF363522 | 11 |
|  | EF363523 | Sao Tomé | EF363523 | 11 |
| *M. schreibersii*  (10) | DQ120915 | Turkey | DQ120915 | 10 |
|  | DQ120914 | Greece | DQ120914 | 10 |
| **Species/clade** | **Specimen label** | **Locality** | **GenBank No.** | **Reference** |
| *M. schreibersii*  (10) | DQ120913 | Southern Iberia | DQ120913 | 10 |
|  | DQ120912 | Southern Iberia | DQ120912 | 10 |
|  | DQ120911 | Southern Iberia | DQ120911 | 10 |
|  | AF376830 | Spain | AF376830 | 16 |
|  | EU360717 | Europe* | EU360717 | 3 |
|  | EU360718 | Europe* | EU360718 | 3 |
|  | EU360719 | Europe* | EU360719 | 3 |
|  | EU360720 | Europe* | EU360720 | 3 |
| *M. australis* (1) | M28191 | Vanuatu | AY614735 | 13 |
| *M. macrocneme* (1) | M19552 | New Guinea | AY614734 | 13 |
| ***M. 'bassanii'* (1) | BAppleton1133 | Australia | AY614733 | 13 |
| ***M. 'orianae'* (1) | BAppleton1137 | Australia | AY614732 | 13 |
| ***M. 'blepotis'* (1) | AF217444 | New Guinea | AF217444 | 2 |
| ***M. 'fuliginosus’*  (3) | AB085735 | Japan* | AB085735 | 17 |
|  | EF530348 | China* | EF530348 | 14 |
|  | EF530346 | China* | EF530346 | 14 |
| *Myotis ricketti* (1) | outgroup | China* | EF530349 | 14 |
